# Cyanamide as a prebiotic phosphate activating agent – catalysis by simple 2-oxoacid salts†
†Electronic supplementary information (ESI) available. See DOI: 10.1039/c7cc07517k


**DOI:** 10.1039/c7cc07517k

**Published:** 2017-10-18

**Authors:** Maria Tsanakopoulou, John D. Sutherland

**Affiliations:** a MRC Laboratory of Molecular Biology, Francis Crick Avenue, Cambridge Biomedical Campus , CB2 0QH , UK . Email: johns@mrc-lmb.cam.ac.uk

## Abstract

Cyanamide is a prebiotically plausible compound that has previously been invoked as a phosphate activating agent. However, its reactions with phosphate monoesters are very slow and tend to be low yielding. We now report a fast and efficient phosphate activation reaction using cyanamide in the presence of glyoxylate or pyruvate. These simple 2-oxoacid salts are shown to function as catalysts and in an optimised system, adenosine-3′-phosphate was converted to adenosine-2′,3′-cyclic phosphate in 95% yield.

## Abstract 2

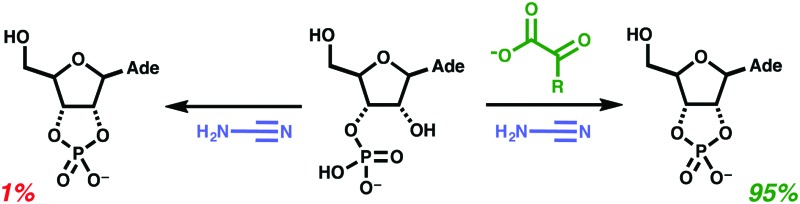
Cyanamide as a fast and efficient prebiotic phosphate activating agent with catalysis by glyoxylate or pyruvate.

Before the advent of biology on the early Earth, the linking of nucleotides to give oligonucleotides must have occurred in a non-enzymatic way. Phosphate activation of some sort was presumably crucial to drive phosphodiester bond formation, a process that is otherwise unfavourable in water. Although we have previously described two prebiotically plausible syntheses of the canonical pyrimidine nucleotides,^1^,^2^ and others have described syntheses of purine nucleotides,^3^,^4^ we still need to uncover efficient prebiotic activation chemistry and energy sources. In this regard, our attention, like that of others over the years, has been drawn to cyanamide **1**, whose fleeting tautomer (HN=C=NH) is reminiscent of the *N*,*N*′-dialkylcarbodiimide condensing agents of conventional synthetic organic chemistry. The feedstocks for the cyanosulfidic chemistry that underpins our nucleotide syntheses have been suggested to arise through thermal metamorphosis of ferrocyanide salts. The products of such metamorphosis are contingent upon the nature of the cation(s) of these salts and heating calcium and magnesium ferrocyanide leads to calcium cyanamide and magnesium cyanamide or nitride (depending on the temperature).^5^–^7^ Hydrolysis of these cyanamide salts produces cyanamide **1** and the metal hydroxide. Other evidence that cyanamide **1** is a plausible prebiotic compound is more circumstantial and is based on the constitutional presence of cyanamide-derivable groupings of atoms in several key biomolecules, and inherently favoured reactions and pathways to such molecules that either incorporate **1**, or are catalysed by derivatives thereof. Thus, cyanamide **1** features large in the prebiotic reaction network that leads to the formation of nucleotides, amino acids and lipid precursors. More specifically, it reacts with glycolaldehyde to form 2-aminooxazole, which is a precursor for the synthesis of nucleotides. It also undergoes phosphate-catalysed hydration to urea, which in turn is a catalyst and medium for phosphorylation reactions. Further, cyanamide **1** reacts with β-aminopropionitrile and β-aminopropionaldehyde en route to arginine.^7^ Accordingly, cyanamide **1** has been thoroughly investigated in the context of phosphate activation, but despite its likely abundance and the favourable thermodynamics of its hydration, it has proven to be too unreactive kinetically. Thus, Orgel found that uridine-3′-phosphate could only be transformed to the 2′,3′-cyclic phosphate derivative in good yields if high concentrations of cyanamide **1** were employed and the reactions were heated for several days (*e.g.* 73% with 800 mM **1**, 1 M acetate buffer, pH 5, 65 °C, 6 days).^8^ Oró showed that treatment with cyanamide **1** led to the prebiotic condensation of mononucleotides, but in very low yields.^9^ Recently, Richert reported that even low level conversion of a nucleoside-5′-phosphate and glycine to the corresponding phosphoramidate required very high concentrations of **1** and extremely long reaction times (75 days).^10^ Given that cyclic nucleotides are labile, we sought conditions under which they could be synthesised far faster than they degrade. This led us to search for catalysts of the activation chemistry. We have now discovered that the activation of monophosphates by cyanamide **1** – as assessed by the conversion of adenosine-3′-phosphate **2** to adenosine-2′,3′-cyclic phosphate **3** – is dramatically accelerated by the prebiotically plausible 2-oxoacid salts, glyoxylate **4** and pyruvate **5**,^11^–^15^ especially so in the presence of magnesium or calcium ions.‡
‡We use numbers to denote compounds regardless of their ionisation state.


At first, cyanamide **1** (to 1 M) was added as a solid to a solution of adenosine-3′-phosphate **2** (50 mM) and glyoxylic acid **4** (10 mM) at room temperature and at different pH values in the range pH 4.5–6.9 (p*K*_a_ of glyoxylic acid **4** ∼3.3), whereupon it was found that pH 5 gave the best yield of adenosine-2′,3′-cyclic phosphate **3** – 11%, according to ^31^P NMR integration, after only 24 h (Scheme 1 and Table 1). This encouraged us because activation by **1** has typically been associated with more prolonged reaction times.

**Scheme 1:**
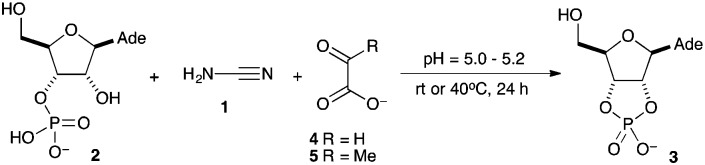
Conversion of **2** to **3** with cyanamide **1** and 2-oxoacid salts **4** or **5**.

**Table 1:** Yield in different conditions for the conversion of **2** to **3** at pH 5

2-Oxoacid salt	Cyanamide **1**	Temperature	Divalent cations (50 mM)	Yield of **3** (%)
**4** (10 mM)	1 M^*a*^	r.t.	—	11
**4** (50 mM)	1 M^*a*^	r.t.	—	23
**4** (50 mM)	1 M^*b*^	r.t.	—	35
**4** (100 mM)	4 × 100 mM^*b*^	r.t.	—	57
**4** (100 mM)	4 × 100 mM^*b*^	40 °C	—	68
**5** (100 mM)	4 × 100 mM^*b*^	r.t.	—	36
**5** (100 mM)	4 × 100 mM^*b*^	40 °C	—	43
**4** (100 mM)	4 × 100 mM^*b*^	r.t.	Mg^2+^	84
**4** (100 mM)	4 × 100 mM^*b*^	40 °C	Mg^2+^	95
**5** (100 mM)	4 × 100 mM^*b*^	r.t.	Mg^2+^	69
**5** (100 mM)	4 × 100 mM^*b*^	40 °C	Mg^2+^	80
**4** (100 mM)	4 × 100 mM^*b*^	r.t.	Ca^2+^	85
**4** (100 mM)	4 × 100 mM^*b*^	40 °C	Ca^2+^	92
**5** (100 mM)	4 × 100 mM^*b*^	r.t.	Ca^2+^	64
**5** (100 mM)	4 × 100 mM^*b*^	40 °C	Ca^2+^	77

^*a*^Fast addition.

^*b*^Slow addition.

We then proceeded to optimise the reaction and found that the yield was significantly improved by increasing the amount of glyoxylate **4**, by slowly adding the cyanamide **1** portionwise and by increasing the temperature to 40 °C (Scheme 1 and Table 1). Pyruvate **5** showed similar reactivity to **4** although yields were slightly lower. Notably, in the absence of the 2-oxoacid salts, **4** or **5**, adenosine-3′-phosphate **2** underwent cyclisation to adenosine-2′,3′-cyclic phosphate **3** in only 1% yield suggesting a significant role for the 2-oxoacid salts in promotion or catalysis. Analysis of the reaction mixture by NMR revealed the presence of glyoxylurea **6** and urea **7** along with the cyclic nucleotide **3** (Scheme 2). Glyoxylurea **6**, which has been studied in depth because of its biological relevance,^16^ exists in equilibrium with urea **7** and glyoxylate **4** and the rate of equilibration is accelerated both by heating and by catalysis by divalent metal cations such as Mg^2+^ and Ca^2+^. Reasoning that availability of glyoxylate **4** in our reaction might be limited by a sluggish breakdown of glyoxylurea **6**, we added divalent metal cations. Yields increased significantly (maximum yield of 95%) bolstering this supposition (Scheme 1 and Table 1).

**Scheme 2:**
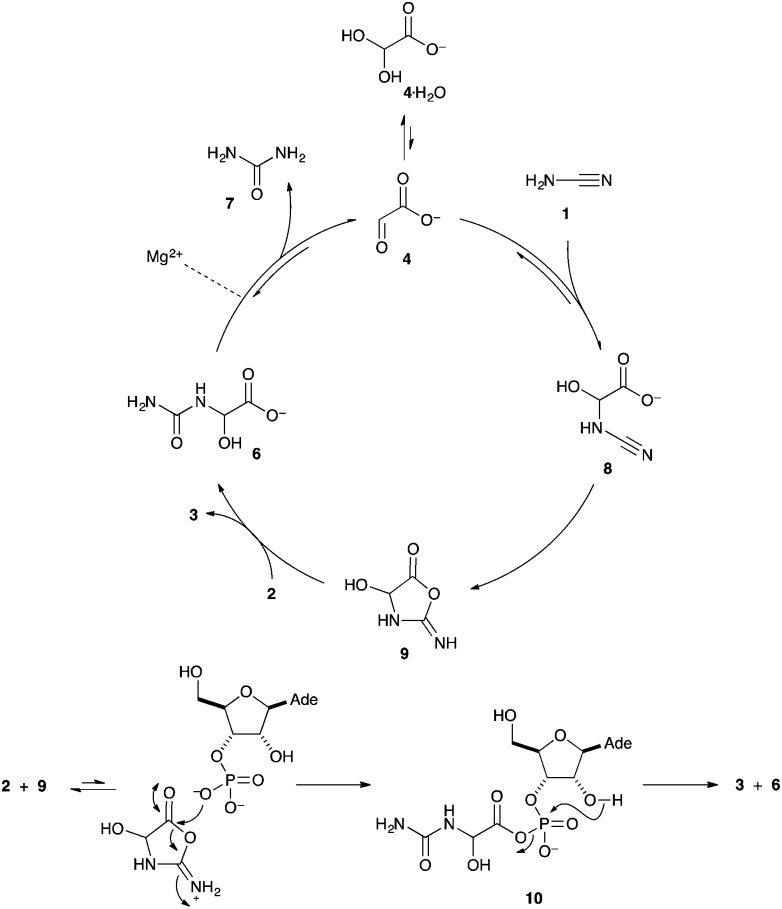
Proposed mechanistic pathway for the reaction in presence of glyoxylate **4**.

Regarding the mechanism of the reaction (Scheme 2), we propose that cyanamide **1** and glyoxylate **4** reversibly associate to give the carbonyl adduct **8** which then cyclises to the *O*-acylisourea **9**. We do not know whether the addition of the glyoxylate carboxyl group to the cyanamide carbon is fast simply due to induced intramolecularity, or because the carbodiimide tautomer of **8** is additionally somehow favoured. As shown in the supplementary information (Fig. S6, ESI†), the reaction displays an initial rapid burst phase and the rate then levels off as an intermediate reaches steady-state. *O*-Acylisourea **9** probably functions as the active intermediate for the phosphate activation, and in so doing is converted to glyoxylurea **6**, which we identified as the steady-state intermediate. As already mentioned, the interconversion of this latter adduct with urea **7** and glyoxylate **4**, is catalysed by divalent metal cations. As regards the actual phosphate activation step, we suspect that the nucleotide monoanion **2** and neutral *O*-acylisourea **9** are in acid–base equilibrium with a more reactive pairing of nucleotide dianion and *O*-acyluronium cation that combine to form the glyoxylurea nucleotide mixed anhydride derivative **10**. Ordinarily, related but simpler mixed anhydrides transfer the acyl group to the 2′-hydroxyl group in preference to cyclisation,^17^ but in this case, the latter reaction appears to predominate forming the cyclised product **3** (Scheme 2).

According to the ^13^C NMR data, cyanamide **1** is consumed as the cyclic nucleotide product **3** is produced. Glyoxylurea **6** is formed in the mixture, but is interconverted in the presence of magnesium cations with glyoxylate **4** and urea **7**. In Fig. 1, the clear conversion of **2** to **3** along with the formation of glyoxylurea **6** and urea **7** can be observed (Fig. 1a–d). It is notable that the intensity of the carbonyl peak of urea **7** at 162.8 *δ* increases as the reaction progresses, thus it can be concluded that the consumption of cyanamide **1** results in the production of urea **7**. In a control experiment, where 1 eq. of cyanamide **1** was added slowly to glyoxylate **4** in the absence of any nucleotide, extensive conversion to glyoxylurea **6** was observed (Fig. 1e). Another control reaction between glyoxylate **4** and urea **7** confirms the equilibration of these two entities with glyoxylurea **6** (Fig. 1f) that has already been reported.^18^ When 2 eq. of cyanamide **1** were quickly added to a solution of glyoxylate **4**, another product in addition to glyoxylurea **6** was formed. This additional product is tentatively assigned as the 5-ureidoacylisourea **11**, which could have resulted from an attack of a second equivalent of cyanamide **1**. This observation perhaps explains why a slow addition of cyanamide **1** is required to produce the 2′,3′-cyclic nucleotide **3** in the highest yields (Scheme 3).

**Fig. 1:**
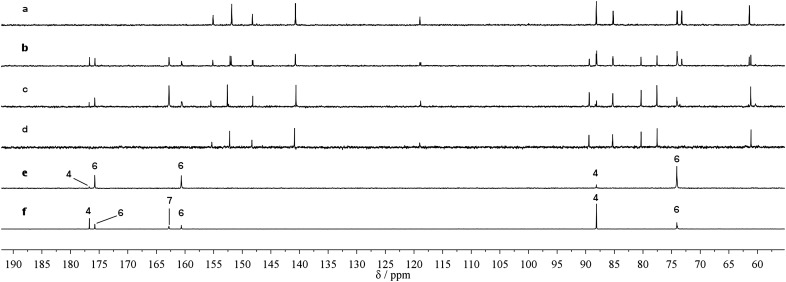
^13^C NMR spectra showing the progress of the reaction in comparison with standards and control experiments; (a) standard sample of adenosine-3′-phosphate **2**, (b) reaction mixture when the yield of cyclisation of **2** was 50%, (c) reaction mixture when the yield of cyclisation of **2** was 90%, (d) standard sample of adenosine-2′,3′-cyclic phosphate **3**, (e) control experiment of glyoxylate **4** + cyanamide **1**, (f) control experiment of glyoxylate **4** + urea **7**.

**Scheme 3:**
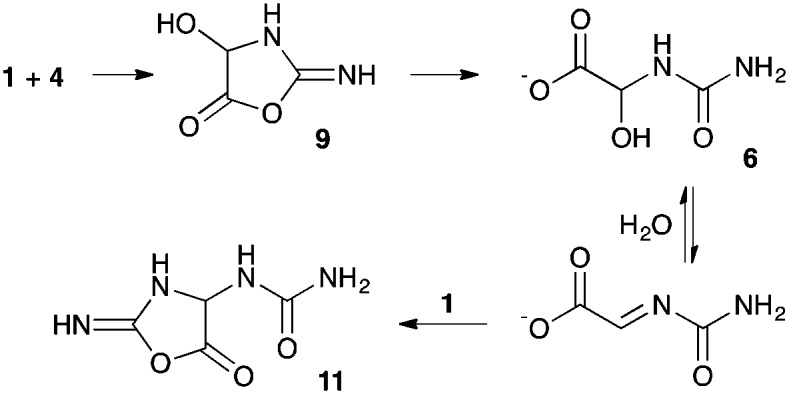
Formation of product **11** when the reaction was performed with fast addition of excess cyanamide **1** to glyoxylate **4**.

The phosphate activation reaction described herein could have taken place on early Earth if a stream containing cyanamide **1** were to have flowed slowly into a body of water containing glyoxylate **4** and nucleotides such as **2**. The enhanced catalysis in the presence of magnesium or calcium ions is intriguing given that our proposed geochemical source of cyanamide **1** involves heating of magnesium and/or calcium ferrocyanide to give the corresponding cyanamide salt followed by carbonation to give a solution of cyanamide **1** and magnesium and/or calcium cations. To further explore this possible geochemical scenario, we repeated the activation reactions of adenosine-3′-phosphate **2** in presence of the 2-oxoacid salts **4** or **5** under optimal conditions (MgCl_2_ and 40 °C), but in this case cyanamide **1** (to 400 mM) was added continuously over 5 h using a syringe pump. The yields for these last reactions are remarkable, since both glyoxylate **4** and pyruvate **5** catalysed the conversion of **2** very efficiently, affording the product adenosine-2′,3′-cyclic phosphate **3** in 92% and 83% yield respectively.

Because of favourable Franck–Condon factors and the strength of its triple bond, the cyanide radical is a likely initial product of high energy chemistry in C, H, O, and N containing atmospheres subject to electrical discharge or impact plasma.^19^ According to a possible multistage scenario, some of this energy could have ended up stored in the form of the triple bond of cyanamide **1**. However, absent catalysis, this triple bond energy is not kinetically accessible to drive the phosphate activation chemistry necessary to produce RNA from nucleotides. Complex enzymatic machinery of the sort biology now employs to produce and utilise activate phosphates would not have been available on the early Earth, but simple 2-oxoacid salts such as glyoxylate **4** and pyruvate **5** could easily have been. Indeed, other authors have described so much other potential chemistry of **4** that the phrase ‘glyoxylate scenario’ has been coined.^20^–^22^ Further given the simplicity and small size of glyoxylate **4**, it is especially noteworthy that it can function as such an effective catalyst for phosphate activation using cyanamide **1**. Over time, the urea **7** that is produced, as cyanamide **1** is consumed by such activation chemistry, would hydrolyse to ammonia and carbon dioxide, gaseous products that would not therefore accumulate in the system. Clean, catalytic nucleotide activation chemistry to drive RNA synthesis *via* nucleoside-2′,3′-cyclic phosphates using energy persisting from violent earlier events might thus have been possible on the early Earth.

This work was supported by the Medical Research Council (No. MC_UP_A024_1009), and a grant from the Simons Foundation (No. 290362 to J. D. S.).

## Conflicts of interest

There are no conflicts to declare.

## Supplementary Material

Supplementary information
