# Supplementary material for: Cyanamide as a prebiotic phosphate activating agent – catalysis by simple 2-oxoacid salts
Source: Chem Commun (Camb). 2017 Oct 18;53(87):11893–6. doi: 10.1039/c7cc07517k (PMC5708344; doi:10.1039/c7cc07517k)
Supplement: Supplementary file 1 [file CC-053-C7CC07517K-s001.pdf]

Electronic Supplementary Material (ESI) for ChemComm.
This journal is © The Royal Society of Chemistry 2017

Supporting Information

Cyanamide as a prebiotic phosphate activating agent – catalysis by simple 2-oxoacid salts

Maria Tsanakopoulou and John D. Sutherland*

MRC Laboratory of Molecular Biology, Francis Crick Avenue, Cambridge Biomedical Campus, CB2 0QH, UK. Email: johns@mrc-lmb.cam.ac.uk

Table of contents

1. General methods S1
2. Reaction of **2** with **1** in the presence of glyoxylate **4** - cyanamide **1** added in one portion S1
3. Reaction of **2** with **1** in the presence of glyoxylate **4** - cyanamide **1** added in four portions S3
4. Reaction of **2** with **1** in the presence of glyoxylate **4** - cyanamide **1** added continuously S6
5. Reaction of **2** with **1** in the presence of pyruvate **5** - cyanamide **1** added in four portions S8
6. Reaction of **2** with **1** in the presence of pyruvate **5** - cyanamide **1** added continuously S9

1. General methods

Adenosine-3′-phosphate **2** (≥98% purity) was purchased from *Sigma-Aldrich* and *Santa Cruz Biotechnology*. All other reagents and solvents were purchased from *Sigma-Aldrich* and *Acros Organics* and were used without further purification. A *Mettler Toledo* SevenEasy pH Meter S20 with a *ThermoFisher Scientific* Orion 8103BN Ross combination semi-micro pH electrode was used to measure and adjust the pH. The syringe pump used was a *Razel* A99-FZ. ^{1}H, ^{31}P, and ^{13}C NMR spectra were acquired using a *Bruker* Ultrashield 400 Plus operating at 400.13, 161.97, and 100.62 MHz respectively. Samples consisting of H_2O/D_2O mixtures were analyzed using HOD suppression to collect ^{1}H NMR spectroscopy data. Chemical shifts (δ) are shown in ppm. The yields of conversion were determined by relative integration of the signals in the ^{31}P NMR spectrum.

2. Reaction of 2 with 1 in the presence of glyoxylate 4 – cyanamide 1 added in one portion

Adenosine-3′-phosphate **2** (8.7 mg, 0.025 mmol) was suspended in water (0.50 mL, containing 10% D_2O) and then glyoxylic acid **4** (12 μL of 0.4 M solution) was added. Glyoxylic acid solution 4 M (120 μL) was prepared by dissolving glyoxylic acid monohydrate (46 mg, 0.5 mmol) in water (90 μL) and it was then diluted to 0.4 M. The pH of the mixture was adjusted to pH 5.0 (Table S1, Entry 3) or to another pH value as stated in Table S1 using 5 M and 1 M NaOH solutions. Cyanamide **1** (21 mg, 0.5 mmol) was then added all at once as a solid. The progress of the reaction at rt was monitored by ^{1}H and ^{31}P NMR and the yields after 24 h are given in the following table according to the ^{31}P integrations (Table S1, Entries 1-7). For the reactions with 50 mM of glyoxylate (Table S1, Entries 8-11), glyoxylic acid **4** (6 μL of 4 M solution) was added to the mixture. For the reaction performed with slow addition of cyanamide (Table S1, Entry 10), the

latter was added dropwise over 10 min using a 100 μL glass syringe, while adjusting the pH of the mixture with 1 M HCl in order to keep it constant in the range pH = 5.0 - 5.2.

Entry	pH	Glyoxylate **4**	Yield of **3**
1	4.5	10 mM	4%
2	4.8	10 mM	10%
3	5.0	10 mM	11%
4	6.0	10 mM	5%
5	6.5	10 mM	2%
7	6.9	10 mM	1%
8	4.0	50 mM	12%
9	5.0	50 mM	23%
10	5.0	50 mM	35%[a]
11	6.0	50 mM	6%

[a] Slow addition of cyanamide.

Table S1 Yields and conditions for the reactions of **2** (50 mM) with cyanamide **1** (1 M) at different pH values and with two different concentrations of glyoxylate **4**.

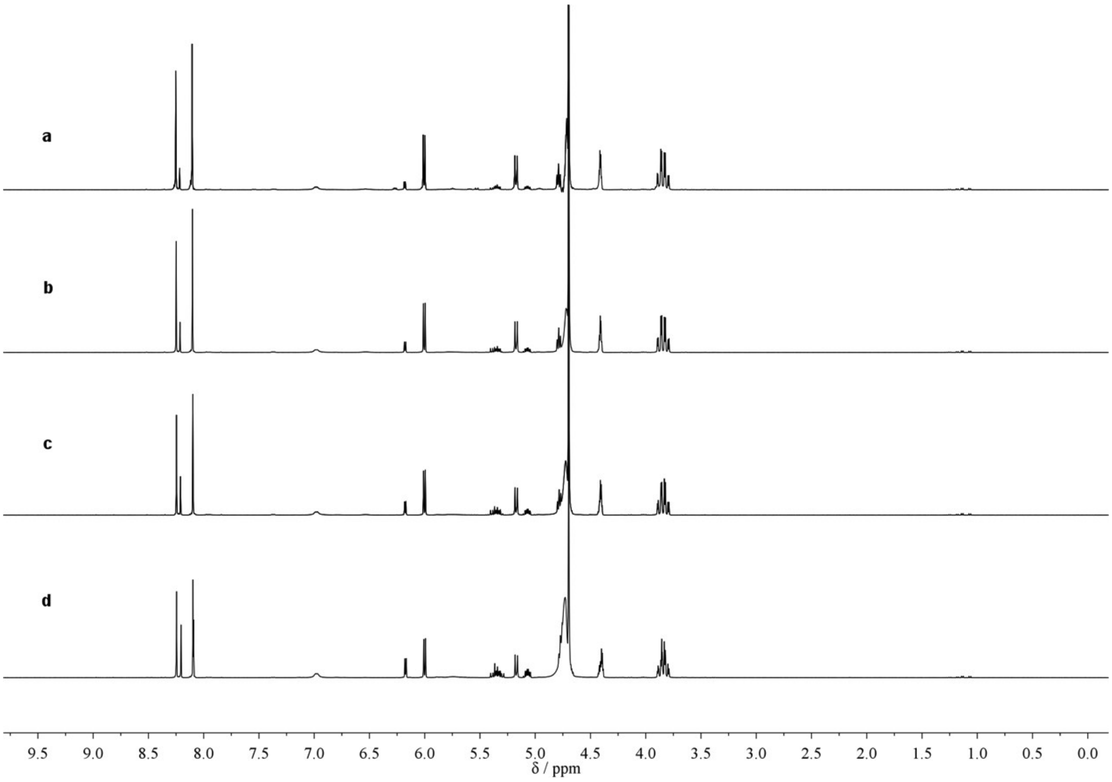

Figure S1 ^{1}H NMR data for the progress of the reaction listed in table S1, entry 10; **(a)** after 10 min **(b)** after 6 h **(c)** after 12 h and **(d)** after 24 h (yield 35%).

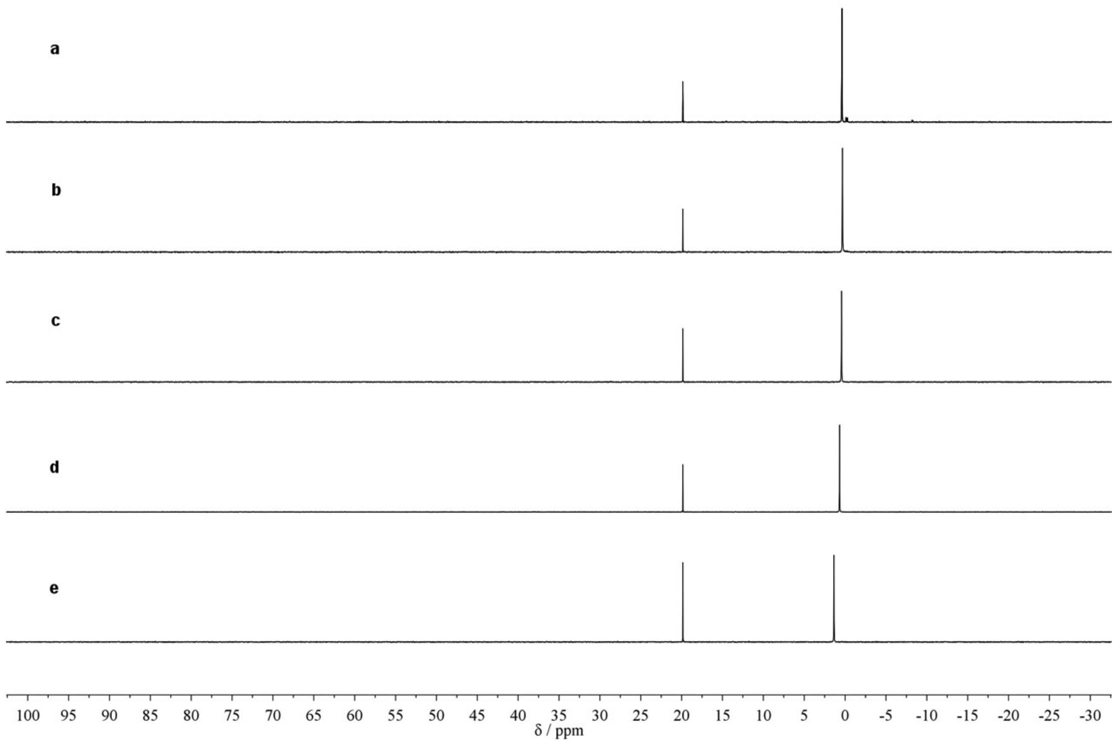

Figure S2 ^{31}P NMR data for the progress of the reaction listed in table S1, entry 10; **(a)** after 10 min **(b)** after 1 h **(c)** after 6 h **(d)** after 12 h and **(e)** after 24 h (yield 35%).

3. Reaction of 2 with 1 in the presence of glyoxylate 4 - cyanamide 1 added in four portions

Adenosine-3′-phosphate **2** (8.7 mg, 0.025 mmol) was suspended in water (0.42 mL, containing 10% D_2O). Glyoxylic acid **4** (12 μL of 4 M solution) was also added to the mixture. Glyoxylic acid solution 4 M (120 μL) was prepared by dissolving glyoxylic acid monohydrate (46 mg, 0.5 mmol) in water (90 μL). In the case of the magnesium catalyzed reaction, magnesium chloride (25 μL of 1 M solution) was added to the mixture. In the other case, where calcium was used, calcium chloride (25 μL of 1 M solution) was added instead. The pH of the mixture was adjusted to 5.0 using 5 M and 1 M solutions of NaOH. Cyanamide **1** (4 x 10 μL of 5 M solution) was added dropwise and very slowly using a 100 μL glass syringe. Cyanamide solution 5 M (200 μL) was prepared by dissolving cyanamide (42 mg, 1 mmol) in water (160 μL). The four additions of 10 μL of cyanamide were performed at certain time points, while adjusting the pH in order to keep it constant in the range pH = 5.0 - 5.2. ^{1}H and ^{31}P NMR data were recorded just before every new addition of cyanamide and also at the end of the reaction at 24 h. There were four time points recorded in total. After the 1st addition of 10 μL of cyanamide, the reaction mixture was left to react at rt or heated at 40°C for 2 h and then NMR data were scanned and pH was adjusted to pH = 5.0 with 1 M HCl before the 2nd addition of cyanamide. The sample was left at rt or heated at 40°C for another 2 h and pH was adjusted before the 3rd

addition. The sample was then left overnight at rt or heated at 40°C for 15 h more and pH was adjusted before the 4th addition. The NMR data were recorded for one more time at the end of the reaction, after another 5 h (24 h in total). Yields for all the experiments are given in table S2.

Time point	Yield at rt	Yield at 40°C	Yield with Mg^{2+} at rt	Yield with Mg^{2+} at 40°C	Yield with Ca^{2+} at rt	Yield with Ca^{2+} at 40°C
1st (2 h)	21%	11%	30%	28%	40%	42%
2nd (4 h)	26%	29%	41%	58%	48%	76%
3rd (19 h)	49%	52%	77%	83%	76%	85%
4th (24 h)	57%	68%	84%	95%	85%	92%

Table S2 Yields for four time points of the reactions of **2** (50 mM) with glyoxylate **4** (100 mM) and cyanamide **1** (4 x 100 mM) under different reaction conditions.

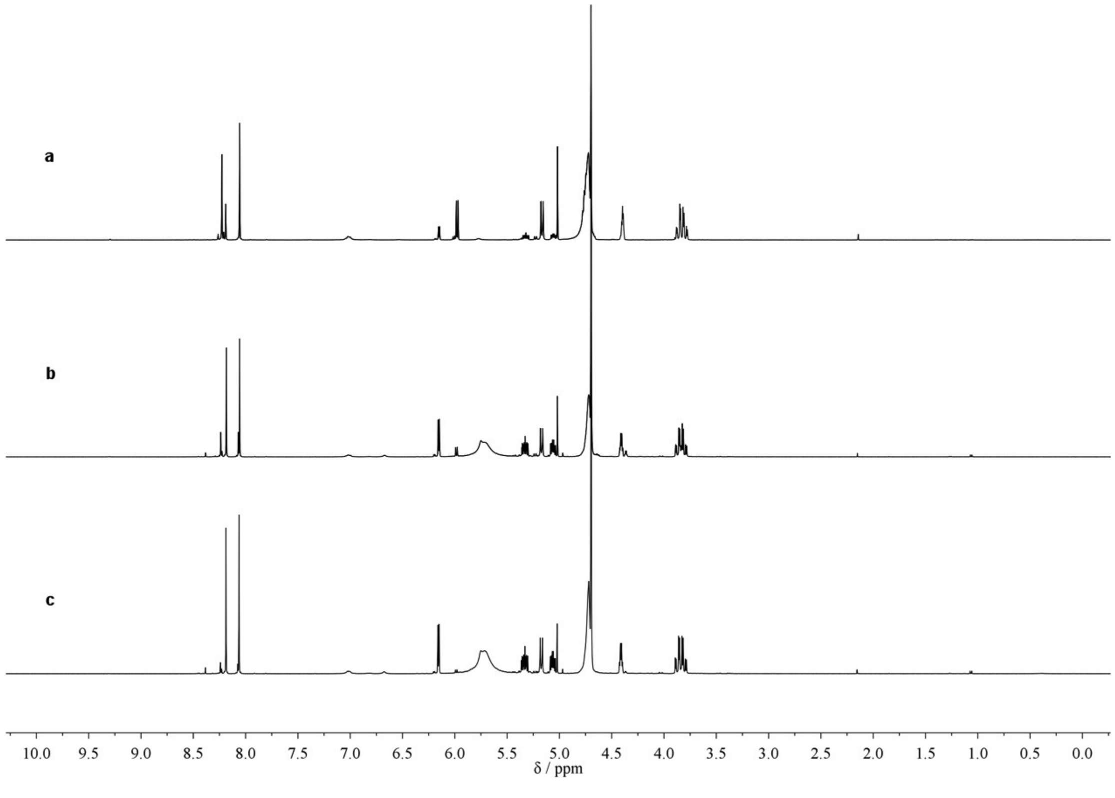

Figure S3 ^{1}H NMR data for the progress of the reaction with Mg^{2+} at 40°C; **(a)** 1st **(b)** 3rd **(c)** 4th time point (yield 95%).

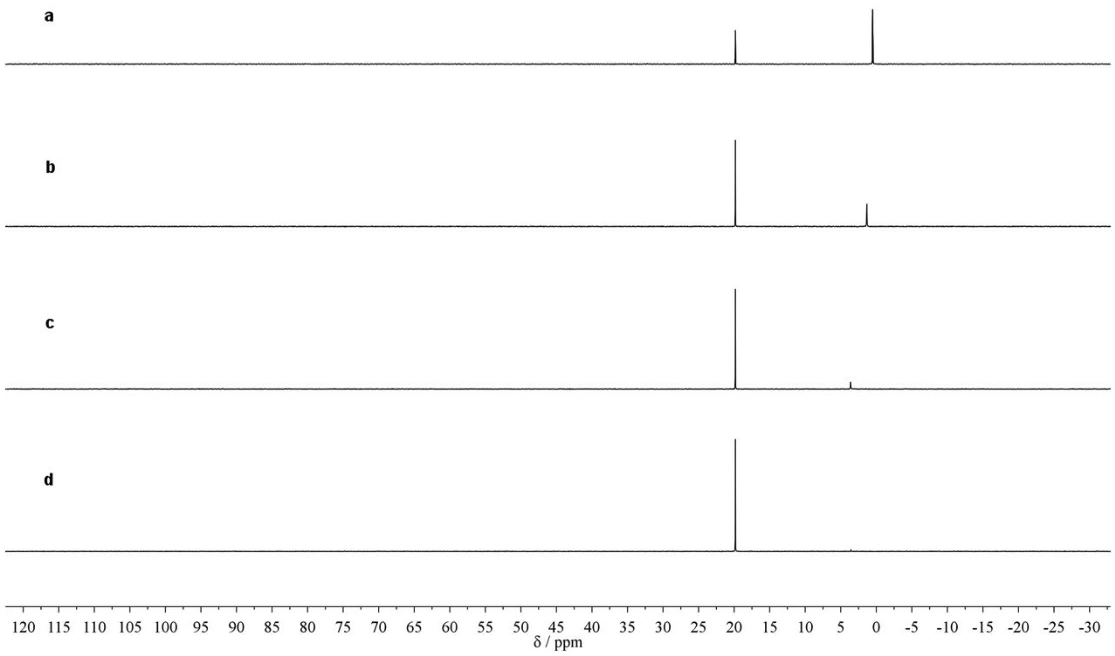

Figure S4 ^{31}P NMR data for the progress of the reaction with Mg^{2+} at 40°C; **(a)** 1st **(b)** 2nd **(c)** 3rd **(d)** 4th time point (yield 95%).

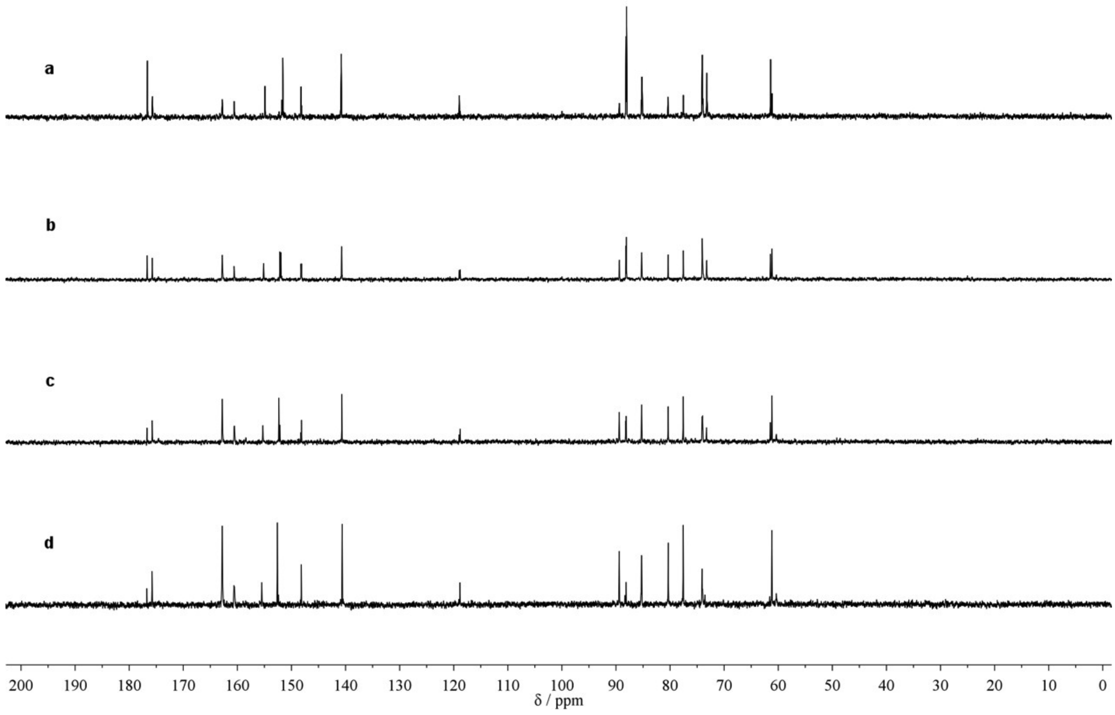

Figure S5 ^{13}C NMR data for the progress of the reaction with Mg^{2+} at 40°C; **(a)** 22% **(b)** 50% **(c)** 70% **(d)** 90% conversion.

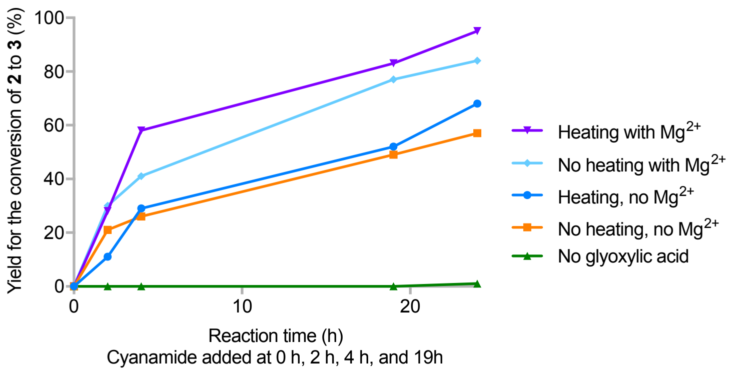

Figure S6 Progress of the reaction of **2** with **1** and **4** with or without Mg^{2+}.

4. Reaction of 2 with 1 in the presence of glyoxylate 4 - cyanamide 1 added continuously

Adenosine-3′-phosphate **2** (17.4 mg, 0.050 mmol) was suspended in water (0.85 mL, containing 10% D_2O) in a 5 mL eppendorf vial. Glyoxylic acid **4** (24 µL of 4 M solution) was added to the mixture. Magnesium chloride (50 µL of 1 M solution) was also added. The pH of the mixture was adjusted to 5.0 using 5 M and 1 M solutions of NaOH. The pH electrode was immersed in the vial together with a small magnetic stirrer. The vial was heated at 40°C in an oil bath and the measuring temperature of the pH meter was set to 40°C. Cyanamide **1** (80 µL of 5 M solution) was added over 5 h using a 100 µL glass syringe and a syringe pump, having the syringe needle immersed into the solution in order to ensure the slowest addition. The pH of the reaction mixture was checked periodically, every 0.5 h and was adjusted in order to be constant in the range pH = 5.0 - 5.2 using 1 M HCl solution. The yield of adenosine-2′,3′-cyclic phosphate **3** was 92%. It has to be noted here that, when the same reaction was performed with syringe pump addition over 20 h, the yield was similar (93%).

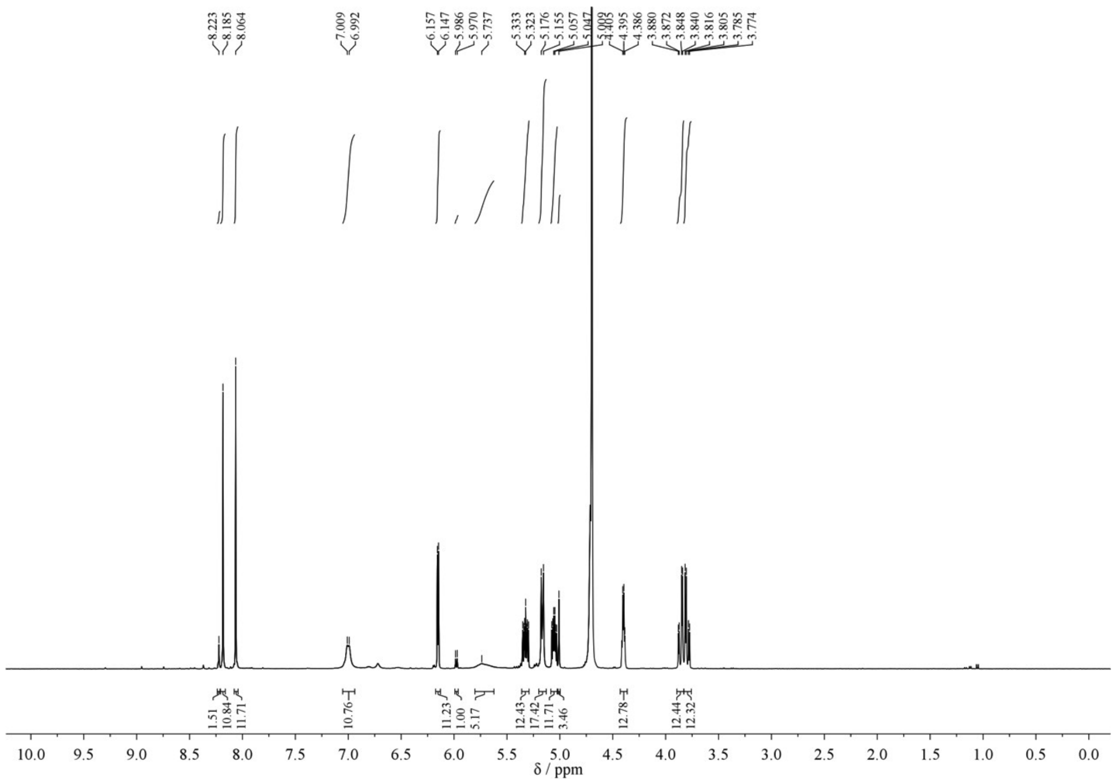

Figure S7 ^{1}H NMR data for the reaction with Mg^{2+} at 40°C and syringe pump addition over 5 h (yield 92%).

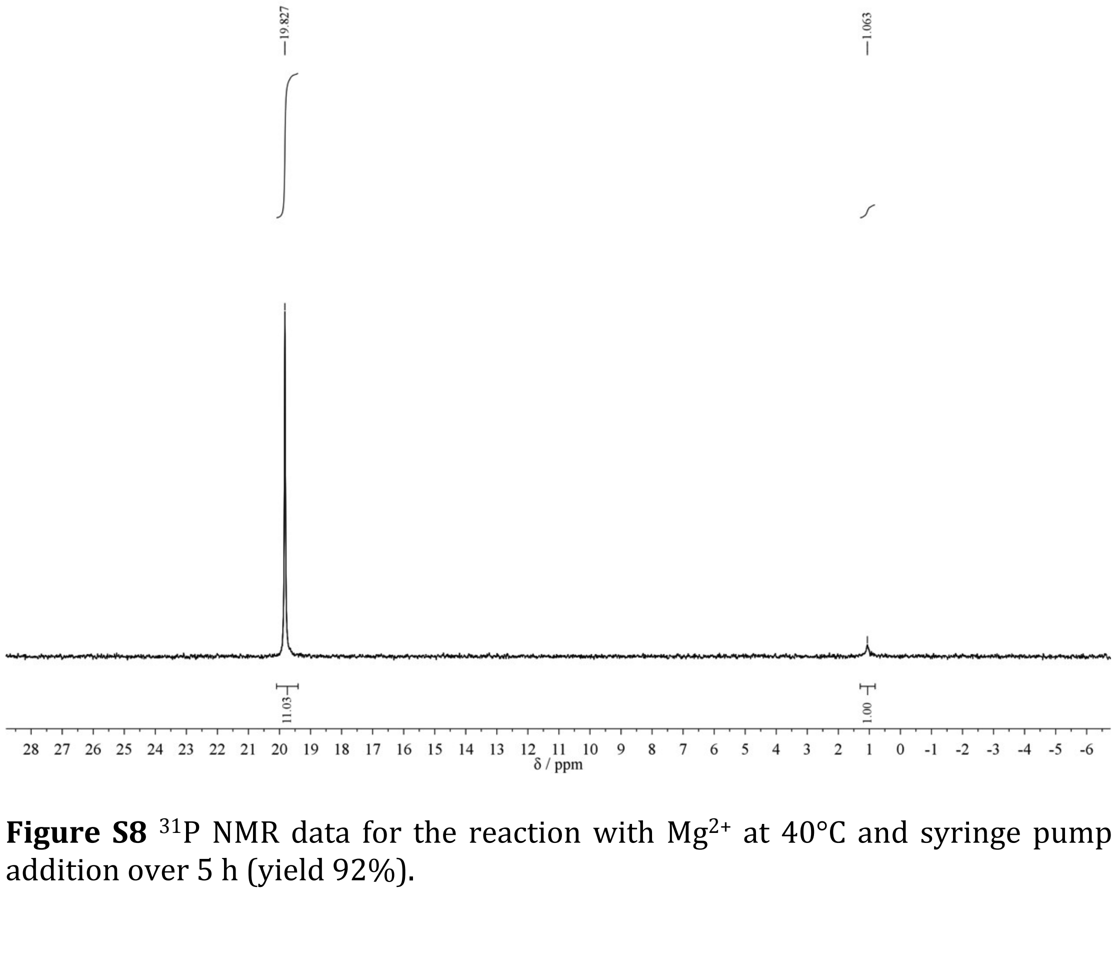

Figure S8 ^{31}P NMR data for the reaction with Mg^{2+} at 40°C and syringe pump addition over 5 h (yield 92%).

5. Reaction of 2 with 1 in the presence of pyruvate 5 - cyanamide 1 added in four portions

The procedure and the amounts used were exactly the same as for the reactions in the presence of glyoxylate **4**. In this case, pyruvic acid **5** (10 µL of 5 M solution) was added to the mixture instead. Pyruvic acid solution 5 M (100 µL) was prepared by dissolving pyruvic acid (44 mg, 0.5 mmol) in water (71 µL). Yields for all the experiments are given in table S3.

Time point	Yield at rt	Yield at 40°C	Yield with Mg^{2+} at rt	Yield with Mg^{2+} at 40°C	Yield with Ca^{2+} at rt	Yield with Ca^{2+} at 40°C
1st (2 h)	12%	15%	19%	21%	29%	31%
2nd (4 h)	25%	29%	42%	47%	47%	55%
3rd (19 h)	35%	41%	56%	66%	58%	63%
4th (24 h)	36%	43%	69%	80%	64%	77%

Table S3 Yields for four time points of the reaction of **2** (50 mM) with pyruvate **5** (100 mM) and cyanamide **1** (4 x 100 mM) under different reaction conditions.

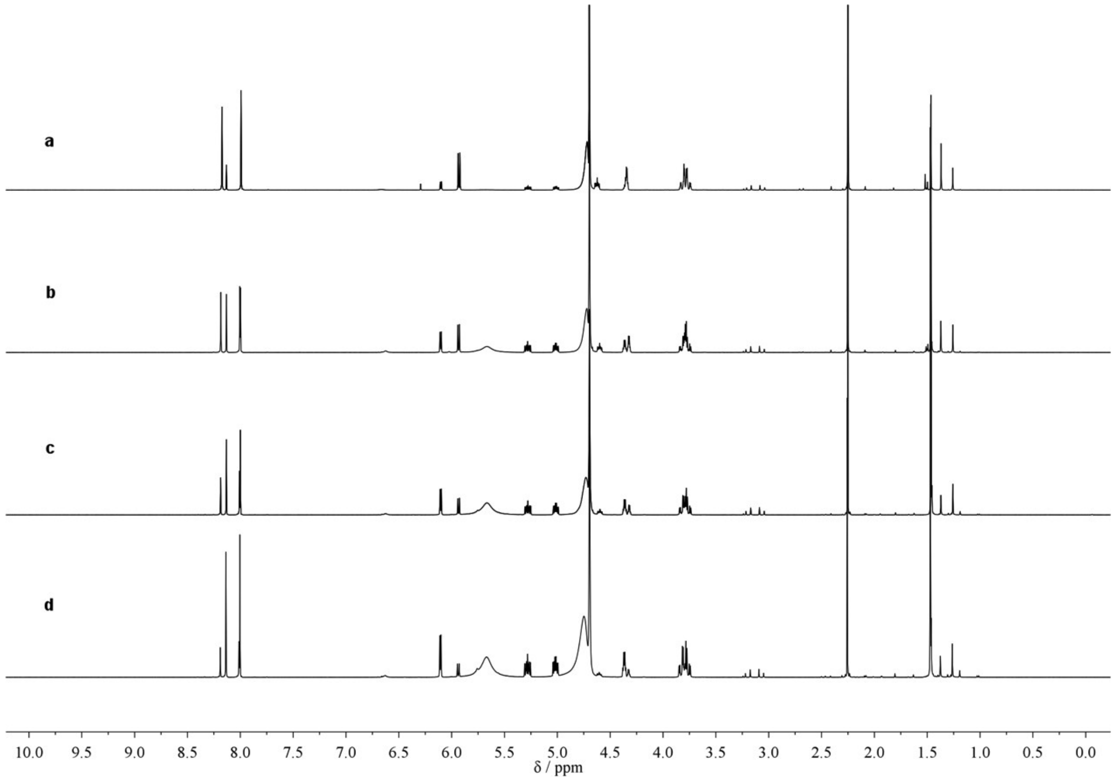

Figure S9 ^{1}H NMR data for the progress of the reaction with Mg^{2+} at 40°C; **(a)** 1st **(b)** 2nd **(c)** 3rd **(d)** 4th time point (yield 80%).

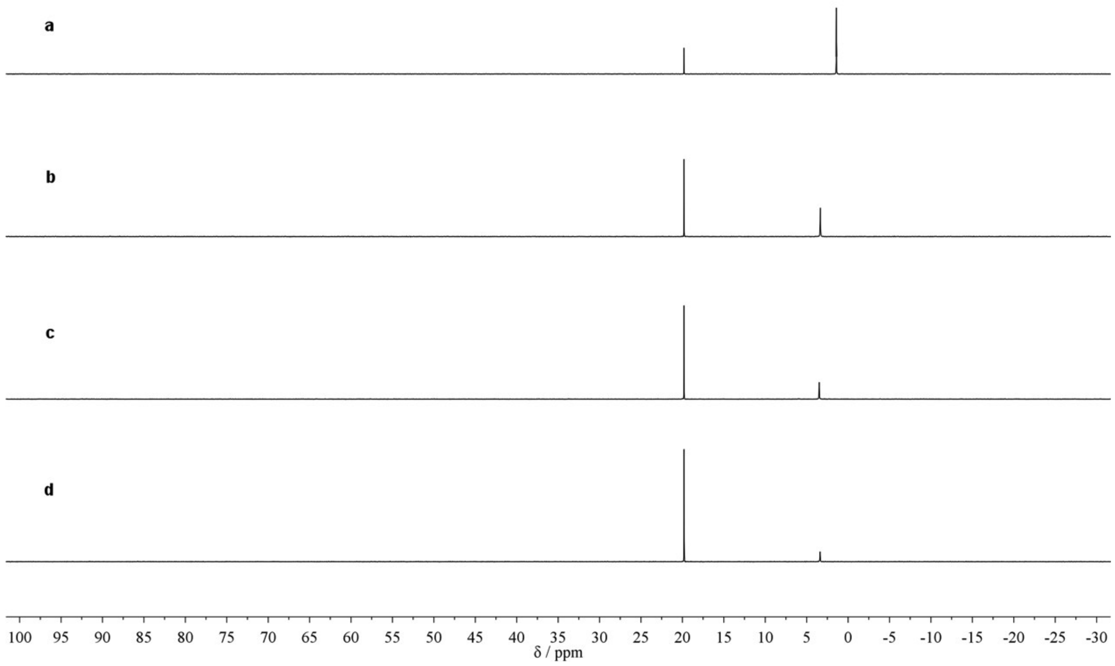

Figure S10 ^{31}P NMR data for the progress of the reaction with Mg^{2+} at 40°C; **(a)** 1st **(b)** 2nd **(c)** 3rd **(d)** 4th time point (yield 80%).

6. Reaction of 2 with 1 in presence of pyruvate 5 - cyanamide 1 added continuously

The procedure and the amounts used were exactly the same as for the reaction in the presence of glyoxylate **4**. In this case, pyruvic acid **5** (20 µL of 5 M solution) was added to the mixture. The yield of adenosine-2′,3′-cyclic phosphate **3** was 83%.

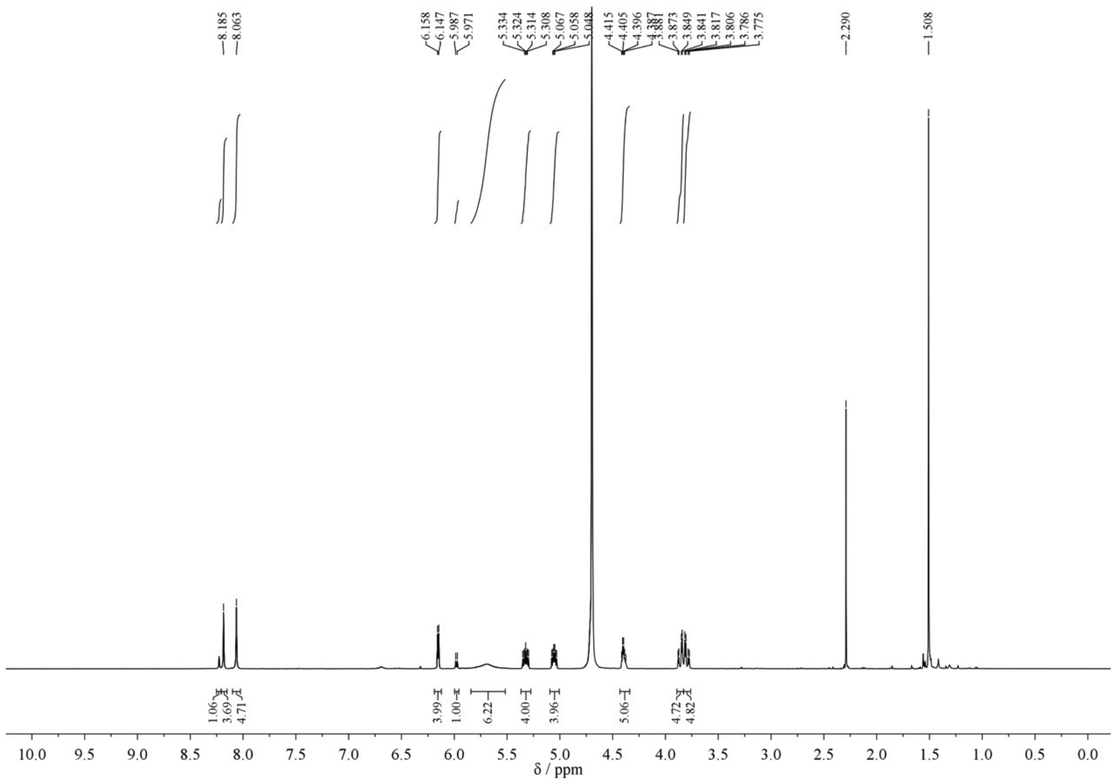

Figure S11 ^{1}H NMR data for the reaction with Mg^{2+} at 40°C and syringe pump addition over 5 h (yield 83%).

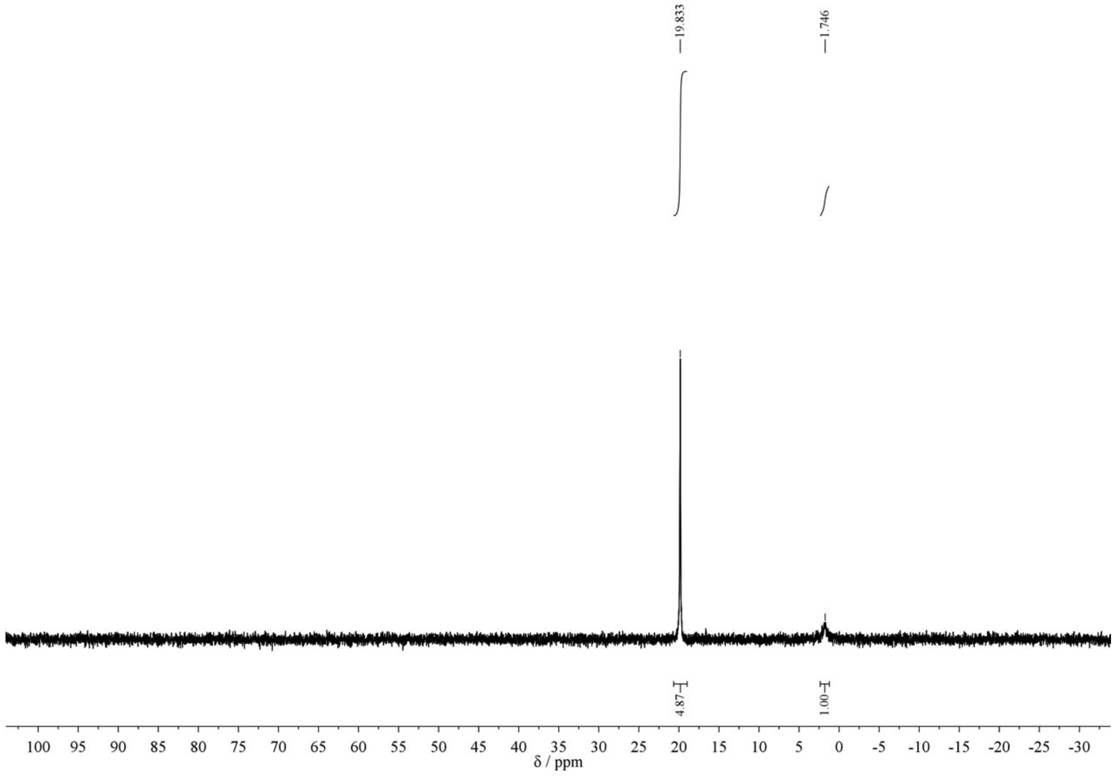

Figure S12 ^{31}P NMR data for the reaction with Mg^{2+} at 40°C and syringe pump addition over 5 h (yield 83%).
